# Easy and efficient production of completely embryonic-stem-cell-derived mice using a micro-aggregation device

**DOI:** 10.1371/journal.pone.0203056

**Published:** 2018-09-19

**Authors:** Kenta Sumiyama, Naomi Matsumoto, Junko Garçon-Yoshida, Hideki Ukai, Hiroki R. Ueda, Yo Tanaka

**Affiliations:** 1 Laboratory for Mouse Genetic Engineering, RIKEN Center for Biosystems Dynamics Research, 1–3 Yamadaoka, Suita, Osaka, Japan; 2 Laboratory for Synthetic Biology, RIKEN Center for Biosystems Dynamics Research, 1–3 Yamadaoka, Suita, Osaka, Japan; 3 Department of Systems Pharmacology, The University of Tokyo, Tokyo, Japan; 4 International Research Center for Neurointelligence (WPI-IRCN), The University of Tokyo, Tokyo, Japan; 5 Laboratory for Integrated Biodevice, RIKEN Center for Biosystems Dynamics Research, 1–3 Yamadaoka, Suita, Osaka, Japan; Macau University of Science and Technology, MACAO

## Abstract

There is an increasing demand for genetically modified mice produced without crossing, for rapid phenotypic screening studies at the organismal level. For this purpose, generation of completely embryonic-stem-cell (ESC)-derived chimeric mice without crossing is now possible using a microinjection or aggregation method with 3i culture medium. However, the microinjection of ESCs into blastocyst, morula, or 8-cell-stage embryos requires a highly skilled operator. The aggregation method is an easier alternative, but the conventional aggregation protocol still requires special skills. To make the aggregation method easier and more precise, here we developed a micro-aggregation device. Unlike conventional 3-dimensional culture, which uses hanging-drop devices for aggregation, we fabricated a polystyrene funnel-like structure to smoothly drop ESCs into a small area (300-μm in diameter) at the bottom of the device. The bottom area was designed so that the surface tension of the liquid-air interface prevents the cells from falling. After aggregation, the cells can be recovered by simply exerting pressure on the liquid from the top. The microdevice can be set upon a regular 96-well plate, so it is compatible with multichannel pipette use or machine operation. Using the microdevice, we successfully obtained chimeric blastocysts, which when transplanted resulted in completely ESC-derived chimeric mice with high efficiency. By changing the number of ESCs in the aggregate, we found that the optimum number of co-cultured ESCs was around 90~120 per embryo. Under this condition, the efficiency of generating completely ESC-derived mice was the same or better than that of the injection method. These results indicated that our microdevice can be used to produce completely ESC-derived chimeric mice easily and with a high success rate, and thus represents a promising alternative to the conventional microinjection or aggregation method, especially for high-throughput, parallel experimental applications.

## Introduction

Mouse genome editing is an indispensable process for organism-level phenotype analysis in biology and medical research. The CRISPR/Cas9 system [1, 2] makes it possible to directly edit the genome of mouse fertilized eggs, generating mutations by homologous recombination (HR) without using embryonic stem cells (ESCs) [3, 4]. The method for directly injecting the CRISPR/Cas9 components into fertilized eggs is easy and efficient for introducing small mutations. Although the use of ESCs is less efficient than the direct introduction of mutations using Cas9 [4, 5], it still has some advantages, such as the ability to generate knock-ins of a relatively large size. Furthermore, knock-in generation by the direct injection method often results in mosaic individuals, which can be problematic for performing phenotype analysis at the F0 generation. Mutant ESCs can be easily maintained and stored without generating mice. Thus, it is possible to retain mutations that cause lethality in later development, and further rescue experiments can be performed in an ESC strain with a lethal mutation [6, 7]. Because of these advantages, ESCs are still used to produce genetically modified mice.

**Fig 1:**
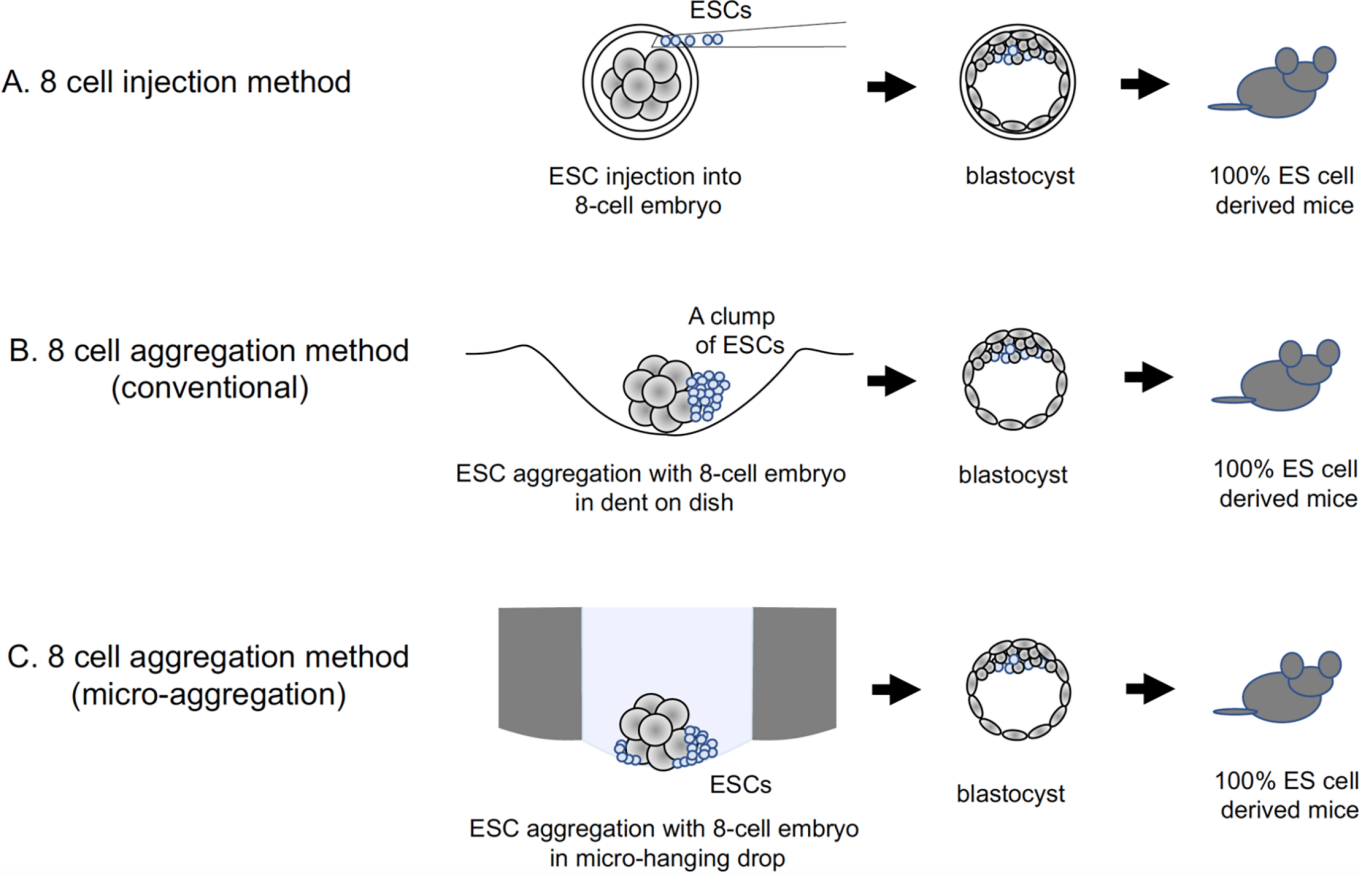
Conventional 8-cell injection method, conventional aggregation method, and newly developed microaggregation method for obtaining 100% embryonic-stem-cell (ESC)-derived mice. (A) Injection method: completely separated ESCs are microinjected into 8-cell-stage embryos. (B) Conventional aggregation method: a clump of ESCs subjected to partial trypsinization are co-cultured with 8-cell-stage embryos with the zona-pellucida removed, in a dent made in the bottom of a culture dish. (C) Microaggregation method: ESCs and 8-cell-stage embryos, both prepared as in B, are separately introduced from the top of the device into a micro-hanging drop and co-cultured. In all three methods, chimeric embryos are cultured until the blastocyst stage and then transplanted into the uterus of recipient mice.

There are several methods for generating individual mice from established ESC lines with targeted mutations. The direct injection of ESCs into blastocysts is commonly used [8]. However, when C57BL/6-lineage ESCs (black coat color) are injected into host embryos such as ICR (white coat color), chimeras can be obtained with different degrees of mosaic coat color. Thus, further crossbreeding is usually necessary to obtain a completely ESC-derived mutant line. Since this process takes nearly a year, it is often the rate-determining step of experiments using knockout or knock-in mice. Recently, improved control of the ESC differentiation state by adding special inhibitors to the culture medium (3i culture method) for ESC establishment and subsequent culture passages, combined with the injection of ESCs into 8-cell-stage embryos rather than blastocysts, enabled the direct generation of completely ESC-derived chimeric mice (Fig 1A) [6, 7]. This procedure makes it possible to analyze mouse phenotypes without crossing, which is highly beneficial for the high-throughput screening of mutation phenotypes on a large scale.

The injection method can produce very good results when a highly skilled operator performs it using a dedicated injection device [6, 7]. However, because it can be difficult to acquire this injection skill and the apparatus is expensive, the injection method cannot be performed in any laboratory. Therefore, as an alternative, a chimeric embryo preparation method using aggregation was established for C57BL/6N ESCs [8–10]. In this procedure, a small indentation is made with a needle on the bottom of a plastic culture dish, and the embryo is brought into contact with a clump of ESCs in the dent (Fig 1B). After incubating this co-culture overnight, embryos containing ESCs are generated. This method produces chimeric mice as efficiently as the injection method. However, it still requires special skills to prepare appropriately shaped dents on the culture dish bottom and to carefully place and recover the embryos and ESCs, to obtain good and reproducible results. It is also difficult to control the number of ESCs that aggregate with the embryo, so it is challenging to determine the optimal quantity of ESCs for the best results. To solve these problems, here we sought to develop a dedicated microdevice for aggregation that could be easily and widely applied to produce completely ESC-derived mice with high reproducibility (Fig 1C).

From the viewpoint of device engineering, a number of reports on cell culture and handling in microfluidic devices have appeared in the past decade or so [11–13]. Most of them are 2-dimensional (2D) culture devices for growing cells on a flat substrate for high-throughput analysis [14]. However, these conditions do not resemble the 3-dimensional (3D) *in vivo* environment [15]. “Body-on-a-chip” or “organ-on-a-chip” 3D-culture systems are recent trends in the “lab-on-a-chip” field [15–18]. To obtain a 3D culture, one major approach is to form spheroids by the air-liquid curved interface in a hanging-drop [19, 20]. Although this method is simple and the recovery is relatively easy, the droplet diameter is more than 1 mm, which results in an air-liquid interface with a relatively large curvature that may not be suitable for the very small number of cells used in ESC–embryo aggregates. To fabricate these large curvature structures, semi-spherical small micro-chamber arrays on a chip have been reported [21, 22]. Although these systems are useful for high-throughput screening, it is difficult to recover the cells after spheroid formation. A system using droplets containing small numbers of cells was also reported [23]; however, in this case the droplet diameter is too small to accommodate large cell masses such as embryos. Thus, in summary, microdevices that can both aggregate cells and recover them well have not yet been realized. To overcome these problems, here we developed a new, very simple micro hanging drop system for mouse ESC-embryo aggregation that is made of polystyrene in 96-well culture-plate format. The fundamental concept of the device compared to conventional methods is summarized in Fig 1.

## Materials and methods

### Animal experiments

ICR mice were purchased from Oriental Yeast Co., Japan. All mice were given food and water ad libitum. Animals were kept in an SPF facility with a 12-hour light and 12-hour dark cycle (lights on at 8:00 am). The ambient temperature was kept around 21 degrees Celsius with a relative humidity of 50%. ICR mice (12 to 20 weeks) were used as recipients. A combination anesthetic (0.75 mg/kg of medetomidine, 4.0 mg/kg of midazolam, and 5.0 mg/kg of butorphanol) was used for surgery. The anesthetics were administered to recipient mice by intraperitoneal injection. All animal experiments were approved by the Institutional Animal Care and Use Committee of the RIKEN Kobe branch (approval number: QA2013-04-4).

### ES cell culture

The ES cell line (R26-H2B-EGFP/mCherry KIES) was established from an F1 mouse generated by crossing R26-H2B-EGFP (CDB0238K) and R26-H2B-mCherry (CDB0239K) provided by LARGE, RIKEN CLST [24]. ES cells were established as described previously [7]. Feeder-free ES cell culture was performed as described previously [25]. Three days after starting the ESC culture, a single-cell suspension of ESCs was prepared as described previously [6]. After centrifugation, the ESC pellet was washed with PBS and re-suspended in a small amount of KSOM medium by gently pipetting several times. The concentration was adjusted with KSOM to 3 x 10^4^ cells per ml, then used for aggregation experiments.

### Mouse embryo preparation and transplantation

The procedures for obtaining, culturing, and transplanting embryos were performed according to standard protocols [8]. Briefly, host ICR embryos were recovered from ICR females at 2.5 days post-coitum and further cultured in KSOM medium until they reached the 8-cell stage. Embryos were treated in acidified Tyrode’s solution (pH 2.5, SIGMA T1788) until the zona pellucida was completely removed, then quickly moved to M2 medium. After aggregation was completed, blastocyst embryos were recovered and transferred into the uterus of pseudopregnant ICR females.

### Device preparation

The device was made by injection molding, a manufacturing process in which parts are produced by injecting material into a mold, following a standard protocol [26], and this procedure was carried out in a manufacturing company (Fujimori Sangyo Co., Ltd, Japan) based on the device design prepared using a 3-dimensional design software (CADmeister, Nihon Unisys, Japan). Briefly, a stainless steel mold was fabricated by precision machining to form the features of the desired part. Then, melted polystyrene at over 170°C was poured into the mold. After cooling to solidify the polystyrene, the final product was removed from the mold. The polystyrene device was then sterilized by a γ beam at 10 kGy. After sterilization, the products were manually set onto each well of a commercially available 96-well polystyrene plate (Nunc) and packed until used for cell culture experiments.

## Results and discussion

### Device design and principle

**Fig 2:**
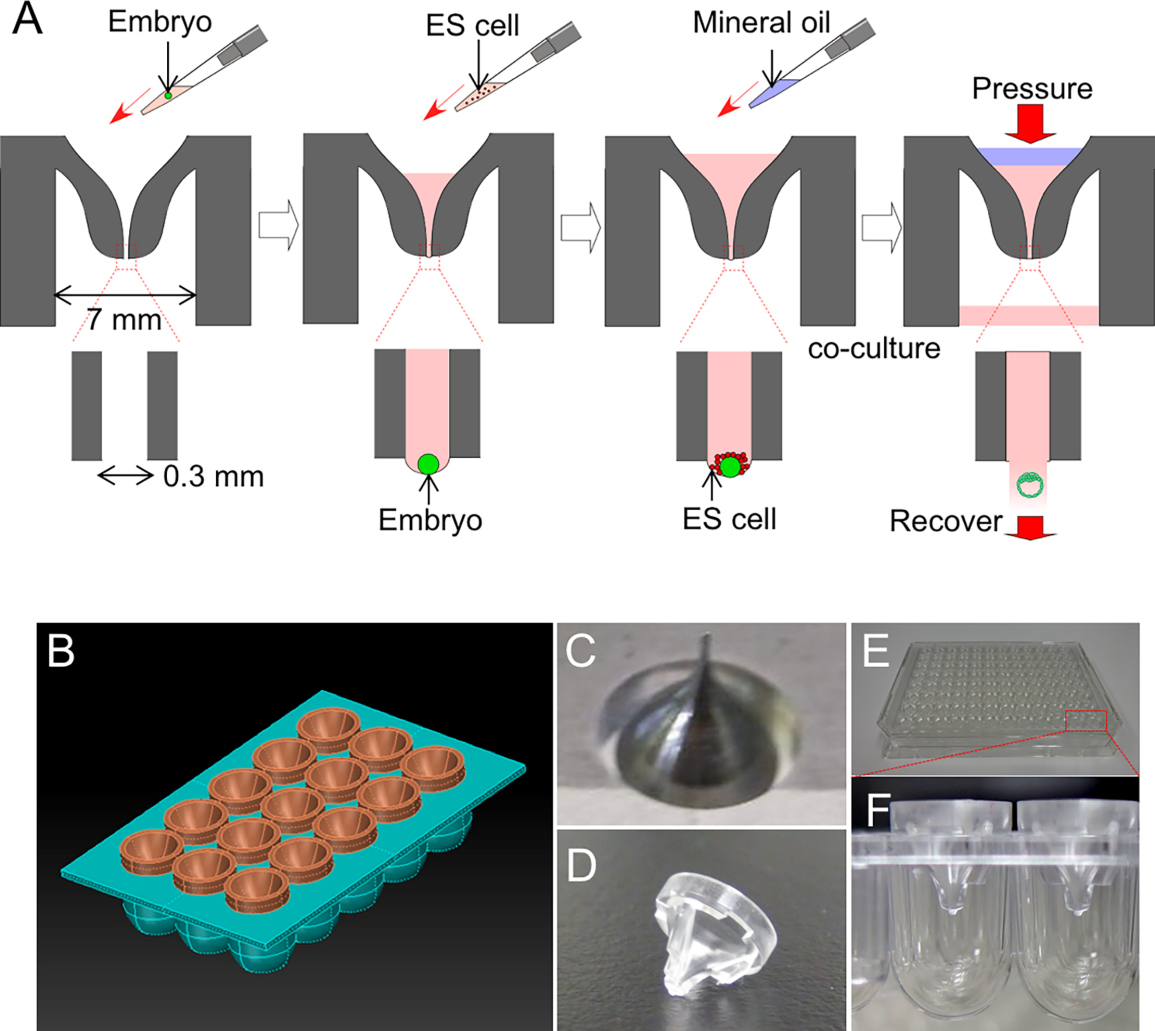
Newly designed device. (A) Cross-sectional schematic view of the cell-aggregation device and the procedure for recovering the ESC-fused embryo using the device. (B) Image of designed devices set on a polystyrene 96-well plate. (C) Metal mold for polystyrene casting. (D) Picture of a fabricated device. (E) A polystyrene 96-well plate equipped with ESC aggregation devices. (F) An enlarged side-view picture of polystyrene devices installed on the 96-well plate.

The microdevice is compatible with a general-purpose 96-well plate, and it can easily be used with multichannel pipetting instruments and machines such as automatic pipetting devices and cell sorters. The device is designed to fit into the wells of a round-bottom 96-well plate. The device is funnel-shaped, with a wide, open upper part that narrows to a capillary at the bottom with an inner diameter of 300 μm (Fig 2A). When the device is filled with medium, aggregation can be performed by co-culturing the embryo and the ESCs at the opening end of the capillary's lower opening, in a minimally sized hanging drop. Because of the steep funnel shape, the introduced ESCs and embryos arrive at the lower part without sticking to the wall, and gather naturally along the curved surface of the liquid bottom (Fig 2A). The number of ESCs introduced can be easily controlled by adjusting the concentration of ESCs in the medium. To prevent evaporation, medium is placed in the well bottom, and the upper part of the device is overlaid with mineral oil (Fig 2A). The device is then placed in an incubator overnight. After incubation, the cells and embryo are collected by pushing the medium downward from the top, using a pipet tip, and collected in the bottom well. It is also possible to recover the aggregates by centrifuging the plate.

The design of the devise on a plate is shown in Fig 2B, and the mold and fabricated devices are shown in Fig 2C–2F.

### Parameter determination

**Fig 3:**
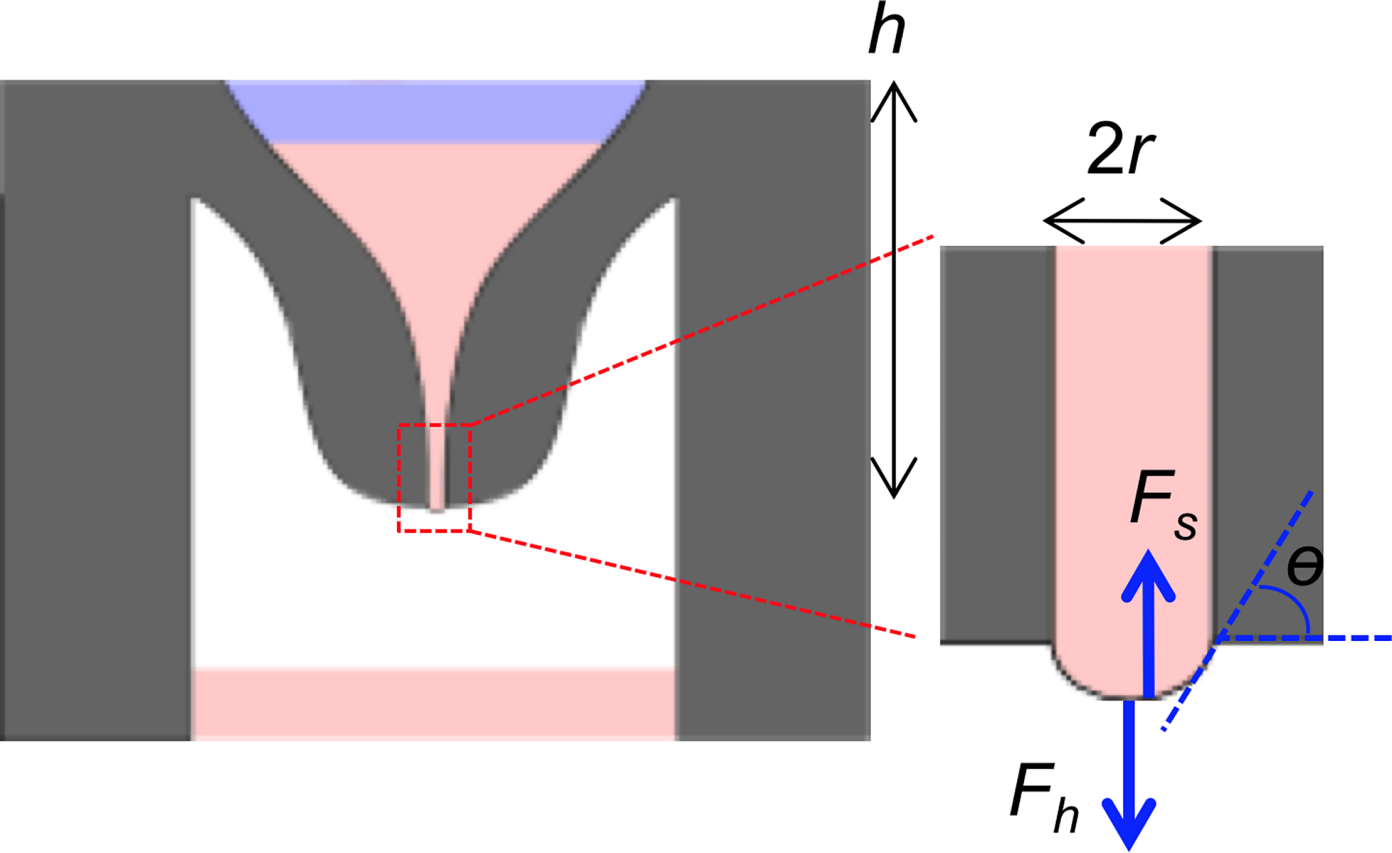
Design parameters used to estimate the gravity and surface tension forces on the medium in the device.

The design of the device was based on the following analysis (also see Fig 3). To maintain the air-liquid interface without losing liquid into the reservoir, the surface tension-based force must be greater than the gravity-based force. The gravity force applied to the medium in the device (*F*_*h*_) was calculated by the following equation:

$$F_{h}=\pi r^{2}\rho gh$$ (Eq 1)

where *r* is the radius of the nozzle of the device, *ρ* is the density of the liquid, *g* is the acceleration rate of gravity, and *h* is the distance from the bottom to the top of the liquid. On the other hand, the surface tension-based force on the medium is calculated by the following equation:

$$F_{s}=2\pi r\gamma \;\cos\;\theta$$ (Eq 2)

where *ɣ* is the surface tension of the liquid and *ɵ* is the contact angle between the liquid surface and the device material (see Fig 3). Eq (1) indicates that *F*_*h*_ is proportional to *r*^*2*^, while Eq (2) indicates that *F*_*s*_ is proportional to *r*. Therefore, as *r* decreases, *F*_*h*_ becomes less than *F*_*s*_. In this calculation, the following physical property values were known: *ρ =* 1.0×10^3^ kg/m^3^, *g* = 9.8 m/s^2^, and *ɣ* = 72 mN/m (around room temperature) [27]. The contact angle (*ɵ*) was determined by pictures of water droplets on the polystyrene material to be 81°±3° (n = 3, error indicates standard deviation). For easy liquid handling and to keep enough medium in the device so that the concentration does not change, the device height (*h*) was designed to be 7.0 mm. Using these values in Eqs (1) and (2), the nozzle radius (*r*) required to make *F*_*h*_ = *F*_*s*_ was calculated to be 0.30 mm. Because some turbulence and vibration would occur during the actual experiments, to ensure that the drop would not fall, *r* was designed to be half the calculated maximum value, namely 0.15 mm.

### Results of aggregation experiments

**Fig 4:**
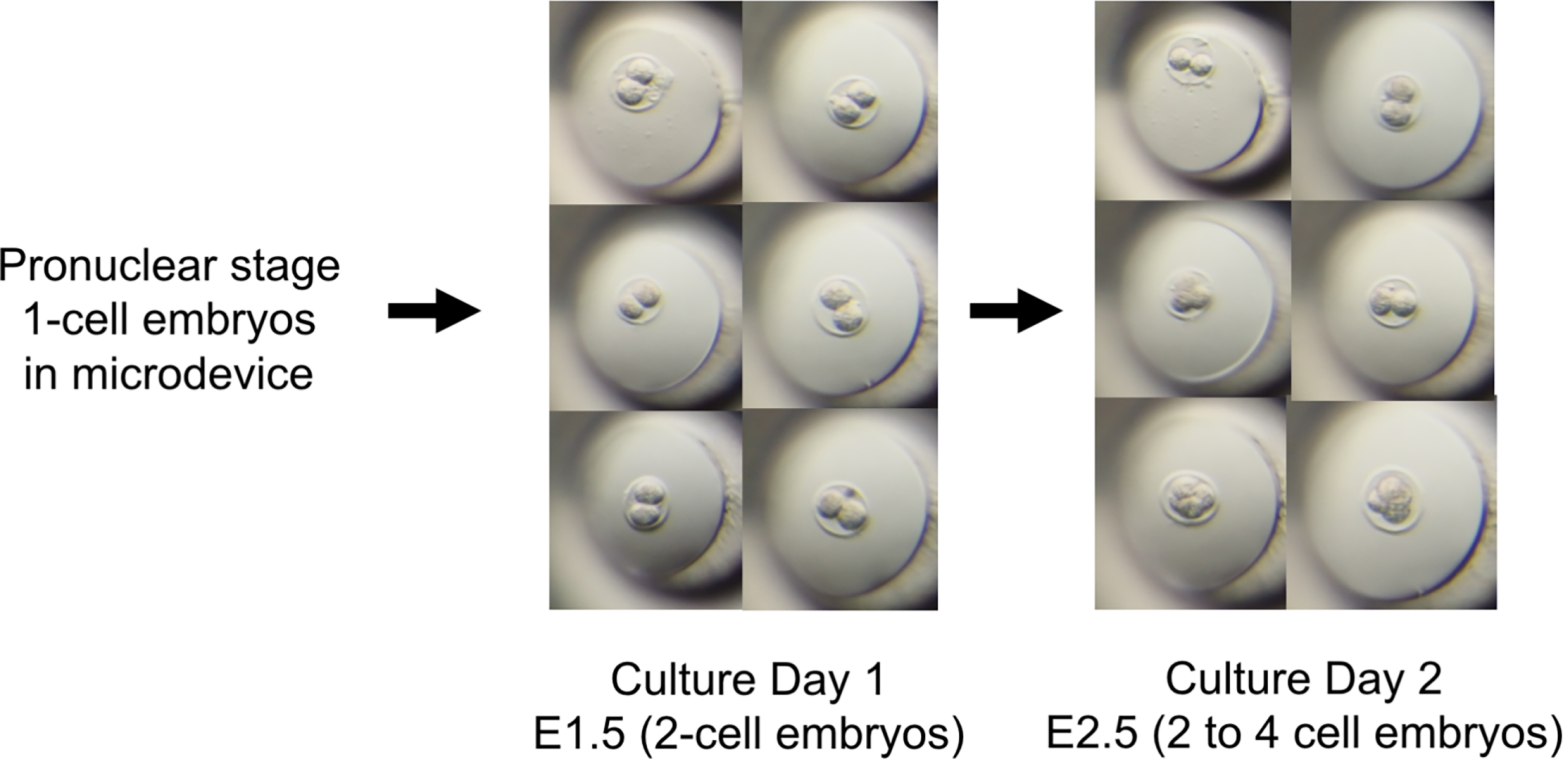
Embryo culture test in the microdevice. Six pronuclear-stage embryos were cultured in the microdevice. After two days of culture, the embryos were alive and had developed normally. Embryos were observed from the top of the microdevice using a stereoscopic microscope.

To confirm that the device is suitable for embryonic culture, pronuclear-stage embryos were first placed in the device filled with KSOM medium and cultured for 2 days. The embryos were alive and developed to the 4-cell stage after incubation. This result indicated that embryos could be safely cultured in the device for at least 2 days (Fig 4).

**Fig 5:**
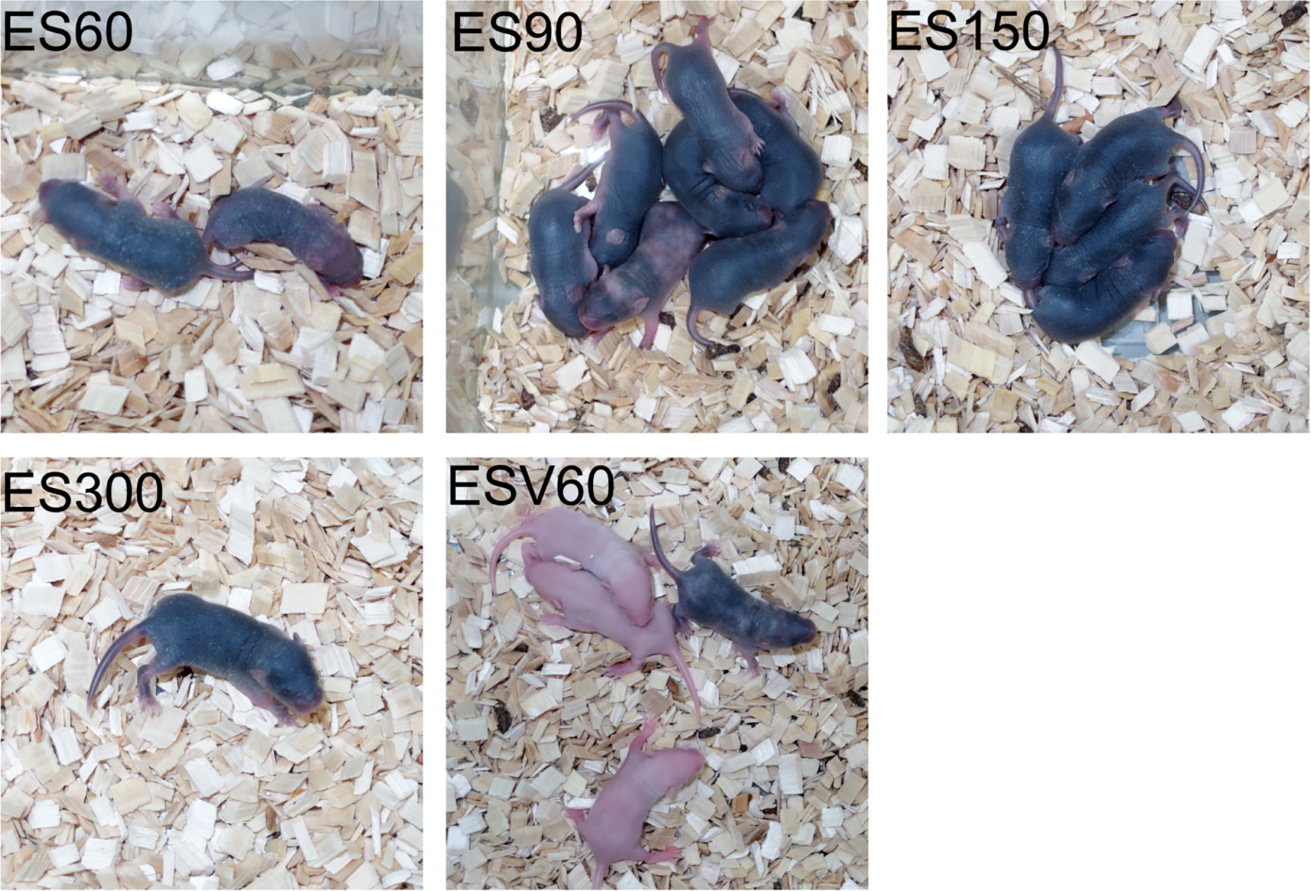
Litters of chimeric founder pups obtained from C57BL/6N-EGFP-mCherry ESCs by microaggregation methods. Pups labeled ES60, ES90, ES150, and ES300 were derived from embryos aggregated in the microdevice with 60, 90, 150, and 300 ESCs, respectively. ESV60 pups were derived from embryos aggregated with 60 ESCs in the bottom of a V-bottomed 96-well culture plate. All pups obtained are pictured except for one ES150 pup that had died.

Next, mouse production experiments using recombinant ESCs (R26-H2B-EGFP / mCherry KIES) were performed. The ESCs were established and maintained in 3i medium (iSTEM medium, Cellartis, Takara Bio, Japan). Eight-cell-stage embryos of ICR mice (Oriental Yeast Co., Japan) were used as the aggregation host embryos. After the device was filled with a small amount of KSOM, a single embryo with the zona pellucida removed was placed in each device, and then ESCs suspended in medium were placed in the device (Fig 2A). The optimal number of ESCs was examined using 60, 90, 150, and 300 cells per well. Twenty-four 8-cell-stage embryos were used for each ESC number condition. After culturing overnight, the embryos were collected from the device. The number of embryos recovered was 20 (60 ESC condition), 22 (90 ESC condition), 24 (150 ESC condition), and 23 (300 ESC condition). The recovered embryos were then transferred into the uterus of recipient ICR pseudopregnant female mice (SLC), and newborns were obtained by Caesarean section. The number of newborn pups obtained from each group was 2 (60 ESC condition), 7 (90 ESC condition), 5 (150 ESC condition), and 1 (300 ESC condition). Among them, 1 (60 ESC condition), 6 (90 ESC condition), 5 (150 ESC condition), and 1 (300 ESC condition) were judged to be completely ESC-derived mice from their coat color (Fig 5). Thus, under each condition we obtained a high proportion of completely ESC-derived mice. Note that not all but just a small fraction of the co-cultured ESCs contributed to the aggregated embryo, given that many ESCs remained outside of the blastocyst when the aggregation was completed. These results showed that the optimum number of ESCs co-cultured per well should be around 90 to 120 per embryo.

To confirm that the ESCs used in the aggregation experiments would also work using a conventional method, we subjected 8-cell-stage embryos (Fig 1A) to injection with the same ESCs as used in the aggregation experiment. Ten ESCs were injected into each 8-cell-stage embryo, and a total of 87 embryos surviving after overnight culture were used for uterus transplantation. The resulting number of newborns was 21, of which 15 were completely ESC-derived mice (Table 1). In this conventional method, we obtained a 71% success rate of completely ESC-derived mice, whereas in the aggregation method using the newly developed microdevice, we achieved 50% to 100% (87% in total), which was comparable to or better than that achieved with the well-established injection method.

**Table 1:** Generation of completely ESC-derived chimeric mice by the microdevice aggregation method.

Method	ESCs	No. of ESCs injected/aggregated per well	No. of Embryos Injected/aggregated and transferred [a]	No. of pups born [b]	No. of completely ESC-derived chimeras [c] (partial chimera)	Ratio [c/b] (c/a)
Microdevice aggregation	R26-H2B-EGFP /mCherry KIES	60	20	2	1 (1)	0.50 (0.05)
Microdevice aggregation		90	22	7	6 (1)	0.86 (0.27)
Microdevice aggregation		150	24	5	5 (0)	1.00 (0.21)
Microdevice aggregation		300	23	1	1 (0)	1.00 (0.04)
Injection		10	87	21	15 (6)	0.71 (0.17)
V-bottom 96-well plate		60	21	4	0 (1)	0 (0)

**Fig 6:**
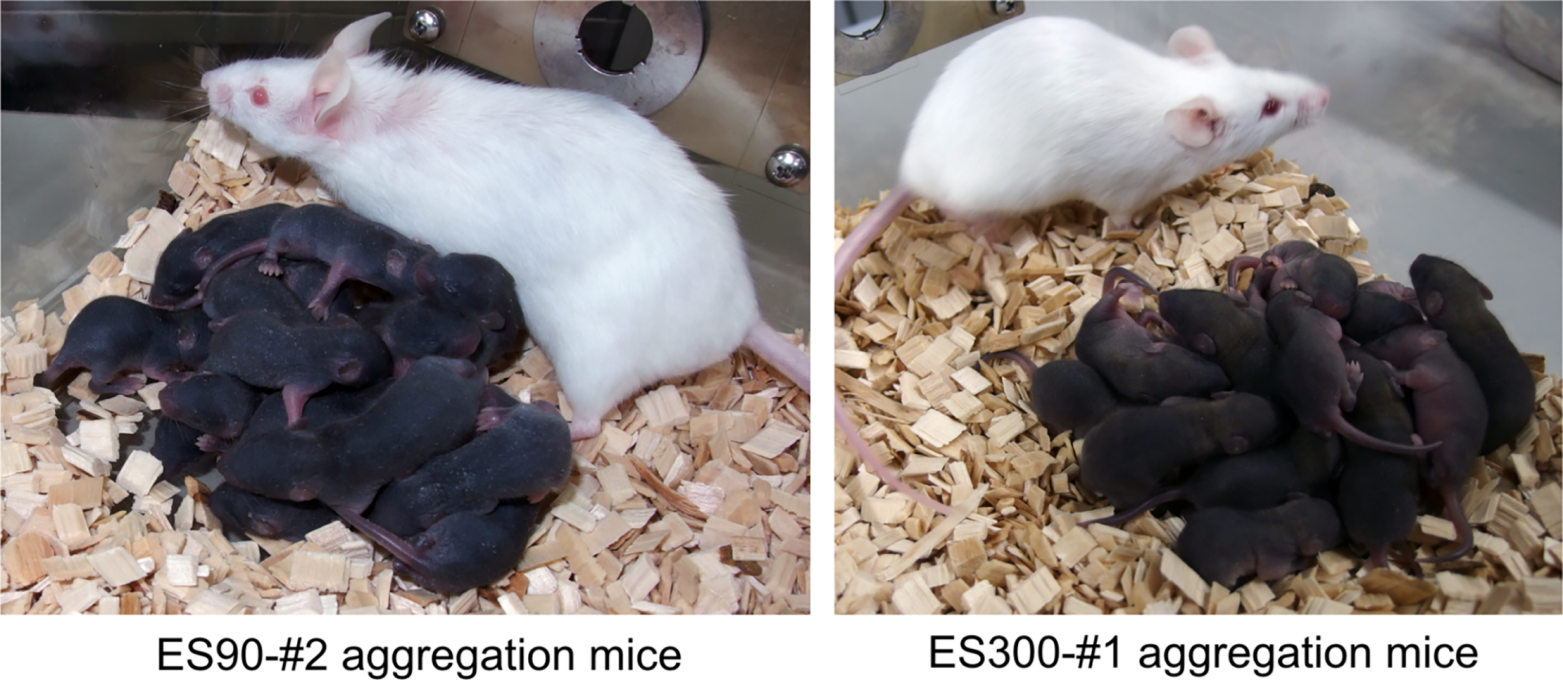
F1 pups obtained by mating a completely ESC-derived male (judged by coat color) with an ICR female (shown in pictures, with a white coat). Left: F1 pups from ID ES90-#2. Right: F1 pups from ID ES300-#1.

To test whether ESC-derived germline cells were transmitted to the next generation, several male mice obtained using the microdevice aggregation method that had a completely black coat were selected and mated with ICR female mice. Notably, all of the resulting newborns had a black coat, indicating successful germline transmission, with no contamination of ICR host cells in the germline (Fig 6, Table 2).

**Table 2:** Germline transmission results of F0 ESC-derived mice with a completely black coat.

ESCs	Mouse line	Mouse ID	No. of pups born (no. of ICR females)	No. of pups with a completely black coat	ESC lineage transmission
R26-H2B-EGFP /mCherry KIES	Aggregation (90 ESCs)	ES90-#2	30 (2)	30	100%
	Aggregation (90 ESCs)	ES90-#4	25 (2)	25	100%
	Aggregation (90 ESCs)	ES90-#5	23 (2)	23	100%
	Aggregation (300 ESCs)	ES300-#1	15 (1)	15	100%

Our results collectively indicated that the aggregation method using the microdevice can produce completely ESC-derived mice with an efficiency comparable to or even better than that of the 8-cell-stage ESC injection method, with a much simpler operation. With this device, researchers can easily produce completely ESC-derived mice without acquiring special operating skills. Furthermore, since a 96-well plate is used, experimental manipulation by a robot can be easily performed, and a high-throughput experimental system can be implemented. That is, combined with automation, this method is scalable and suitable for high-throughput operations.

We also compared this system with larger devices designed to produce conventional embryoid bodies. First, the hanging drop culture plate for preparing the embryoid body was excluded from consideration, because the size of the drop was much larger and the curvature of the liquid surface was too low to enable the ESCs and the embryo to aggregate well. Second, we examined a commercially available V-bottom 96-well culture plate. We tested and confirmed that embryoid bodies could be prepared in this plate, and then co-cultured 60 ESCs and an 8-cell-stage embryo in each well. Of 24 co-cultures performed, 21 embryos were obtained after overnight culture, and uterus transplantation was performed. From these embryos, four newborns were obtained, but all of them were chimeras with a low ESC contribution, as indicated by a white or mosaic coat color (Table 1, Fig 5). This result showed that the production of ESC-derived mice by aggregation using a conventional large-sized culturing apparatus with a low-curvature bottom surface is not very efficient. In contrast, our newly developed micro-aggregation device with a micro-hanging drop system is broadly applicable and highly effective for producing completely ESC-derived mice with ease.

## Supporting information

S1 Checklist The ARRIVE guidelines checklist. Completed “The ARRIVE Guidelines Checklist” for reporting animal data in this manuscript.
(PDF)
